# X-ray phase-contrast computed tomography visualizes the microstructure and degradation profile of implanted biodegradable scaffolds after spinal cord injury

**DOI:** 10.1107/S160057751402270X

**Published:** 2015-01-01

**Authors:** Kenta Takashima, Masato Hoshino, Kentaro Uesugi, Naoto Yagi, Shojiro Matsuda, Atsushi Nakahira, Noriko Osumi, Masahiro Kohzuki, Hiroshi Onodera

**Affiliations:** a Department of Internal Medicine and Rehabilitation Science, Tohoku University Graduate School of Medicine, Sendai, Japan; b Department of Electrical and Electronic Engineering, University of Tokyo, Tokyo, Japan; c Japan Synchrotron Radiation Research Institute, SPring-8, Hyogo, Japan; d R&D Department, Gunze Limited, Shiga, Japan; e Department of Materials Science, Graduate School of Engineering, Osaka Prefecture University, Osaka, Japan; f Division of Developmental Neuroscience, Tohoku University Graduate School of Medicine, Sendai, Japan

**Keywords:** X-ray phase-contrast CT, Talbot grating interferometer, biodegradation, tissue engineering, spinal cord injury

## Abstract

Tissue engineering strategies for spinal cord repair are a primary focus of translational medicine after spinal cord injury (SCI). Many tissue engineering strategies employ three-dimensional scaffolds, which are made of biodegradable materials and have microstructure incorporated with viable cells and bioactive molecules to promote new tissue generation and functional recovery after SCI. It is therefore important to develop an imaging system that visualizes both the microstructure of three-dimensional scaffolds and their degradation process after SCI. Here, X-ray phase-contrast computed tomography imaging based on the Talbot grating interferometer is described and it is shown how it can visualize the polyglycolic acid scaffold, including its microfibres, after implantation into the injured spinal cord. Furthermore, X-ray phase-contrast computed tomography images revealed that degradation occurred from the end to the centre of the braided scaffold in the 28 days after implantation into the injured spinal cord. The present report provides the first demonstration of an imaging technique that visualizes both the microstructure and degradation of biodegradable scaffolds in SCI research. X-ray phase-contrast imaging based on the Talbot grating interferometer is a versatile technique that can be used for a broad range of preclinical applications in tissue engineering strategies.

## Abstract 2

X-ray phase-contrast computed tomography imaging based on the Talbot grating interferometer is described, and the way it can visualize the polyglycolic acid scaffold, including its microfibres, after implantation into the injured spinal cord is shown.

## 1. Introduction   

Loss of neuronal function after spinal cord injury (SCI) remains a devastating condition. Clinical treatments are generally limited to reduction of tissue edema and prevention of secondary injuries through the administration of anti-inflammatory drugs such as methylprednisolone (Bracken, 2012 ▶). However, advances in tissue engineering are opening possibilities that extend beyond symptomatic relief to include neuroprotection and neurorepair after SCI (Pashuck & Stevens, 2012 ▶). Rapid progress is being made in the design of biocompatible materials for tissue engineering, and the development of biopolymers and biodegradable grafts for surgical implantation after SCI holds promise for therapeutic use. Many tissue engineering strategies employ three-dimensional scaffolds made of biocompatible materials that are incorporated with viable cells and bioactive molecules to promote the generation of new tissue and functional recovery (Schmidt & Leach, 2003 ▶; Subramanian *et al.*, 2009 ▶; Straley *et al.*, 2010 ▶; Volpato *et al.*, 2013 ▶). These three-dimensional scaffolds have microstructure such as pores (Thomas *et al.*, 2013 ▶), grooves (Goldner *et al.*, 2006 ▶), capillary (Prang *et al.*, 2006 ▶) or polymer fibres (Yao *et al.*, 2009 ▶; Hurtado *et al.*, 2011 ▶) to support orderly aligned cells and promote directed axonal growth, allowing reconstruction of the neuronal network. In addition, they are often fabricated from biodegrade polymers such as polyglycolic acid (PGA), polylactic acid and their copolymers to reduce chronic inflammations after implantation. Visualization of the microstructure of the scaffolds during the degradation process is essential to track their success. The focus of future SCI research will be on imaging that visualizes both the microstructure and degradation of scaffolds.

X-ray absorption-contrast micro-computed tomography (CT) imaging and magnetic resonance imaging (MRI) have been used to image three-dimensional scaffolds in biological research studies. However, MRI often requires contrast enhancement and it is difficult to visualize three-dimensional microstructure of scaffolds due to limitations in temporal and spatial resolution (Prang *et al.*, 2006 ▶). Similarly, with micro-CT imaging, the contrast of polymeric scaffolds is often too low to extract information on scaffold microstructure when scanned in the tissue or a watery environment such as culture medium (van Lenthe *et al.*, 2007 ▶). In addition, these techniques cannot quantify the degradation rate of the polymeric scaffolds after implantation in the injured spinal cord. X-ray phase-contrast CT imaging with a radiant light source has great potential to allow the visualization of tissue microstructure and mass density without needing a contrast agent. X-ray phase-contrast CT imaging techniques are based on the phase shift of X-ray waves that occurs due to refraction and have approximately a thousand-fold higher sensitivity for detecting light elements, such as hydrogen, carbon, nitrogen and oxygen, than conventional X-ray absorption-contrast techniques (Momose *et al.*, 1996 ▶). In fact, X-ray phase-contrast CT imaging has successfully visualized the microstructure of normal and pathological tissues including brain (Connor *et al.*, 2009 ▶), kidney (Wu *et al.*, 2009 ▶), artery (Shinohara *et al.*, 2008 ▶), blood vessel (Hu *et al.*, 2012 ▶) and eye (Hoshino *et al.*, 2011 ▶, 2012 ▶). In addition, the phase factor in the complex refractive index, on which this imaging contrast is based, is proportional to mass density (Momose *et al.*, 1996 ▶; Hoshino *et al.*, 2011 ▶). Therefore, X-ray phase-contrast CT images can map the variation in mass density of implanted scaffolds in the soft tissue as well as visualize the microstructure.

The purpose of this study was to examine the feasibility of using X-ray phase-contrast CT imaging to visualize the microstructure and quantify the density alteration of scaffolds implanted in the injured spinal cord. We describe the X-ray phase-contrast CT technique and show that it can visualize the microstructure of a scaffold, including the hollow structure and PGA microfibres, after implantation into an injured spinal cord. Furthermore, the X-ray phase-contrast CT images revealed that the braided scaffold gradually degraded from the end to the centre of the scaffold in the 28 days after implantation into the injured spinal cord. This report provides the first demonstration of an imaging technique that visualizes both the microstructure and degradation process of scaffold in SCI research.

## 2. Materials and methods   

### 2.1. Preparation of braiding scaffolds   

The scaffolds were fabricated using a circular braiding machine (R&D Department of Gunze Limited, Shiga, Japan). Single PGA fibres (10 µm in diameter) were braided into a scaffold with a density of eight yarns per bundle around a centrally located metal wire (100 µm in diameter). The diameter of the braided scaffold was 120–130 µm, and scaffolds were stored in a desiccated box. On the day of implantation the metal wire was removed from the braided scaffold to form a channel with a diameter of 100 µm.

### 2.2. Surgical procedures   

**Figure 1:**
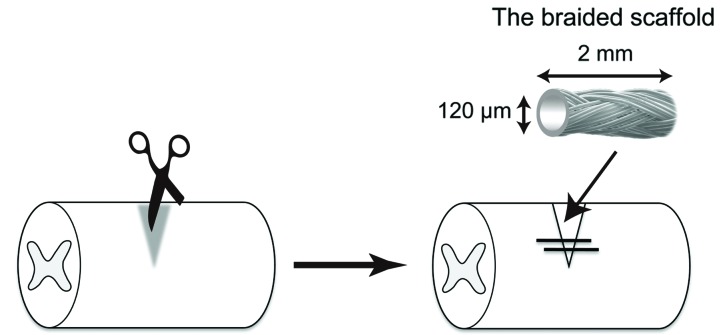
Schematic representation of implantation of the braided scaffolds into the injured spinal cord. Braided scaffolds were fabricated by interweaving eight PGA microfibres (10 µm in diameter) and have a hollow tubular structure (approximately 120–140 µm in diameter). Spinal cord injury (SCI) was made by bilateral transection of the dorsal spinal cord at the tenth thoracic level. Immediately after SCI, animals were implanted with two braided scaffolds (2 mm long) bridging the injured area (dorsal column).

Adult female Sprague Dawley rats weighing 180–220 g were used. All surgical procedures were performed under deep isoflurane anesthesia. The spinal cord was exposed at the tenth thoracic level and a lesion was made with micro-scissors by lowering the scissors from the spinal cord surface by 2 mm to cut the spinal cord bilaterally while keeping the anterior spinal artery and the ventral part of the spinal cord intact. Immediately after SCI, animals were implanted with two scaffolds (2 mm long) bridging the injured area (dorsal column) (Fig. 1 ▶). The muscles and skin were closed in layers after the operation. Rats were allowed to survive for 7 or 28 days after SCI and then perfused. All animal experiments were approved by the Tohoku University Committee for Animal Experiments (Approval No. 2011idou342) and were carried out in strict accordance with the guidelines of the experimental animal care committee of Tohoku University School of Medicine as well as the National Institutes of Health (NIH), USA. The number of animals in this study was kept to a minimum and, when possible, all animals were anesthetized to minimize their suffering during surgery or sacrifice. Animals had access to food and water *ad libitum* and were kept under a 12 h light–dark cycle.

### 2.3. X-ray phase-contrast imaging   

To analyze the degradation of implanted scaffolds, SCI animals implanted with scaffolds were allowed to survive for 7 (*n* = 3) or 28 days (*n* = 4) after implantation. As a control, braided scaffolds were placed on fixed normal spinal cord on day 0. Animals were deeply anesthetized with sodium pentobarbital and perfused with 4% paraformaldehyde in phosphate buffered saline. Spinal cords were incubated in the same fixative at 277 K until CT imaging. X-ray phase-contrast CT imaging based on the X-ray grating interferometer was performed at the Synchrotron Radiation Facility (Japan Synchrotron Radiation Research Institute, SPring-8, Hyogo, Japan), as reported previously (Hoshino *et al.*, 2011 ▶, 2012 ▶). Briefly, the X-ray grating interferometer, constructed at the bending-magnet beamline BL20B2 at SPring-8, utilizes a monochromatic X-ray beam that is passed through a Si(111) double-crystal monochromator. The X-ray energy was 25 keV. The Talbot grating interferometer has two transmission gratings: a phase grating (G1) and an absorption grating (G2). G1 was made of tantalum and G2 was made of gold; the pattern thicknesses were 2.1 µm and 16.6 µm, respectively. The grating pitch of both gratings was 10 µm, and the pattern area size was 25 mm (H) × 25 mm (V). G2 was inclined by 45° to increase the effective X-ray absorption at the grating. An appropriate X-ray imaging detector (consisting of a beam monitor and a charge-coupled device camera) was selected to acquire an image of the whole sample with an adequate field of view. In this measurement the effective pixel size and field of view were 11.7 µm and 23.4 mm (H) × 15.4 mm (V), respectively. Phase retrieval was achieved using a fringe-scan method. G2 was shifted with a motorized stage, and five-step fringe-scans were used. Differential phase shift images were obtained and integrated to provide the phase shift image. The spinal cords were attached to a brass rod that was suspended in saline (1.006 g cm^−3^) within a specially constructed cell. The phase shift was calibrated to perform quantitative analyses of specimens.

The following equation was used to convert the phase shift value per pixel (ΔΦ) to the X-ray refractive index difference from saline in the cell (Δδ),
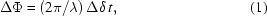
where λ is the X-ray wavelength and *t* is the pixel size of the X-ray detector. The δ value in the complex refractive index is proportional to the density of the sample; therefore Δδ can be used to quantify the density distribution of the sample. The X-ray phase-contrast CT images mapped the variation in Δδ, where Δδ is the difference between the refractive index of the tissue and that of saline at 25 keV X-ray energy. To quantify scaffold degradation, regions of interest were defined by manually tracing the outline of scaffolds on coronal X-ray phase-contrast CT images using *ImageJ* software (National Institutes of Health, Bethesda, MD, USA). The mean Δδ value was calculated in each region of interest. To evaluate the difference in density across the scaffold, the scaffolds were divided into three portions. The Δδ values of each portion of the scaffold were evaluated from six coronal X-ray phase-contrast CT images per scaffold. Δδ is approximately proportional to mass density (ρ) (Momose *et al.*, 1996 ▶; Hoshino *et al.*, 2011 ▶, 2012 ▶), which is estimated from Δδ with the following formula: ρ = (Δδ + δ_w_)/δ_w_ where the refractive index of water (w) is 1 − δ_w_ (δ_w_ = 3.69 × 10^−7^).

### 2.4. Histology   

Histology was performed according to previous reports (Maekawa *et al.*, 2005 ▶; Matsumoto *et al.*, 2011 ▶). Four weeks after implantation, animals were deeply anesthetized with pentobarbital sodium (Kyoritsu Seiyaku Co., Tokyo, Japan) by intraperitoneal injection and perfused with 4% para­formaldehyde dissolved in phosphate buffered saline. Fixed tissue was cryoprotected in 20% sucrose in phosphate buffered saline, embedded and frozen at 193 K in optimum cutting temperature compound (Sakura Finetechnical, Tokyo, Japan), and cryosectioned (25 µm thick). Frozen sections were washed with Tris-based saline containing 0.1% Tween-20. Images of prepared sections were obtained with a microscope (LSM510 Meta; Carl Zeiss, Jena, Germany).

### 2.5. Statistical analysis   

All statistical tests were performed with the *JMP* statistical software program (SAS Institute, Cary, NC, USA). A one-way analysis of variance was performed to compare the density at 0, 7 and 28 days after implantation. If groups were statistically different in the analysis of the variance test, further *post hoc* analyses were carried out using the Tukey–Kramer method for comparisons between groups. A Student’s t-test was performed to compare the density in the central and end portions of the scaffold. For all statistical analyses, significance was set at *p* < 05. Data are presented as mean ± standard error of the mean (SEM).

## 3. Results   

### 3.1. Visualization of the braided scaffold in the injured spinal cord using X-ray phase-contrast CT imaging   

**Figure 2:**
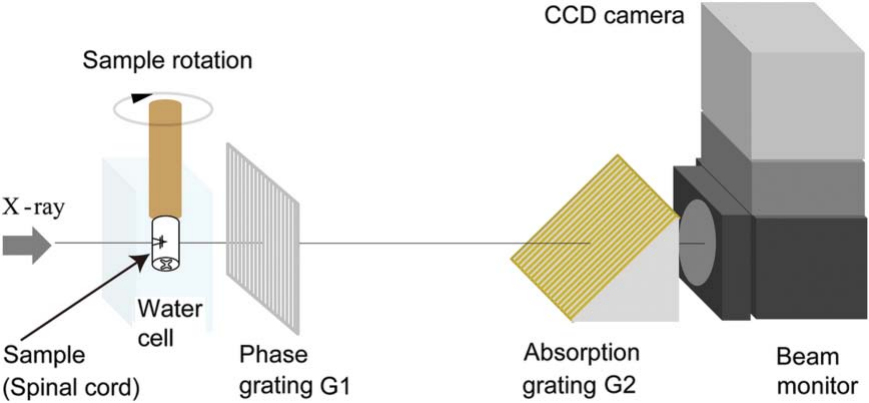
The experimental setup for X-ray phase-contrast imaging based on the grating interferometer. The sample is soaked in a cell filled with saline that has windows through which the X-rays can pass.

**Figure 3:**
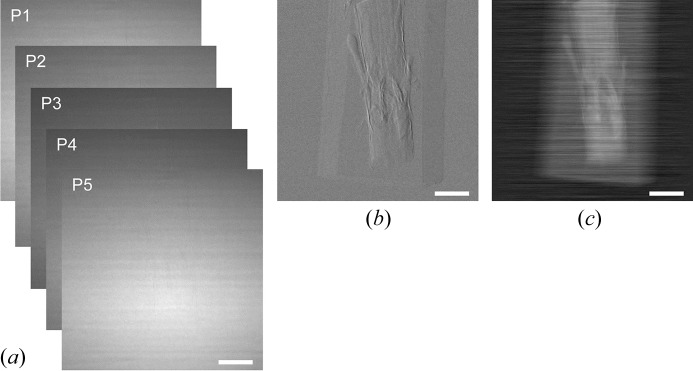
X-ray phase-contrast images of spinal cord after scaffold implantation. (*a*) Raw images of the spinal cord after scaffold implantation observed with the X-ray grating interferometer. (*b*) Differential phase-shift image obtained by a fringe-scan calculation. (*c*) Phase-shift image obtained by one-dimensional integration of (*b*). Scale bar = 2 mm.

**Figure 4:**
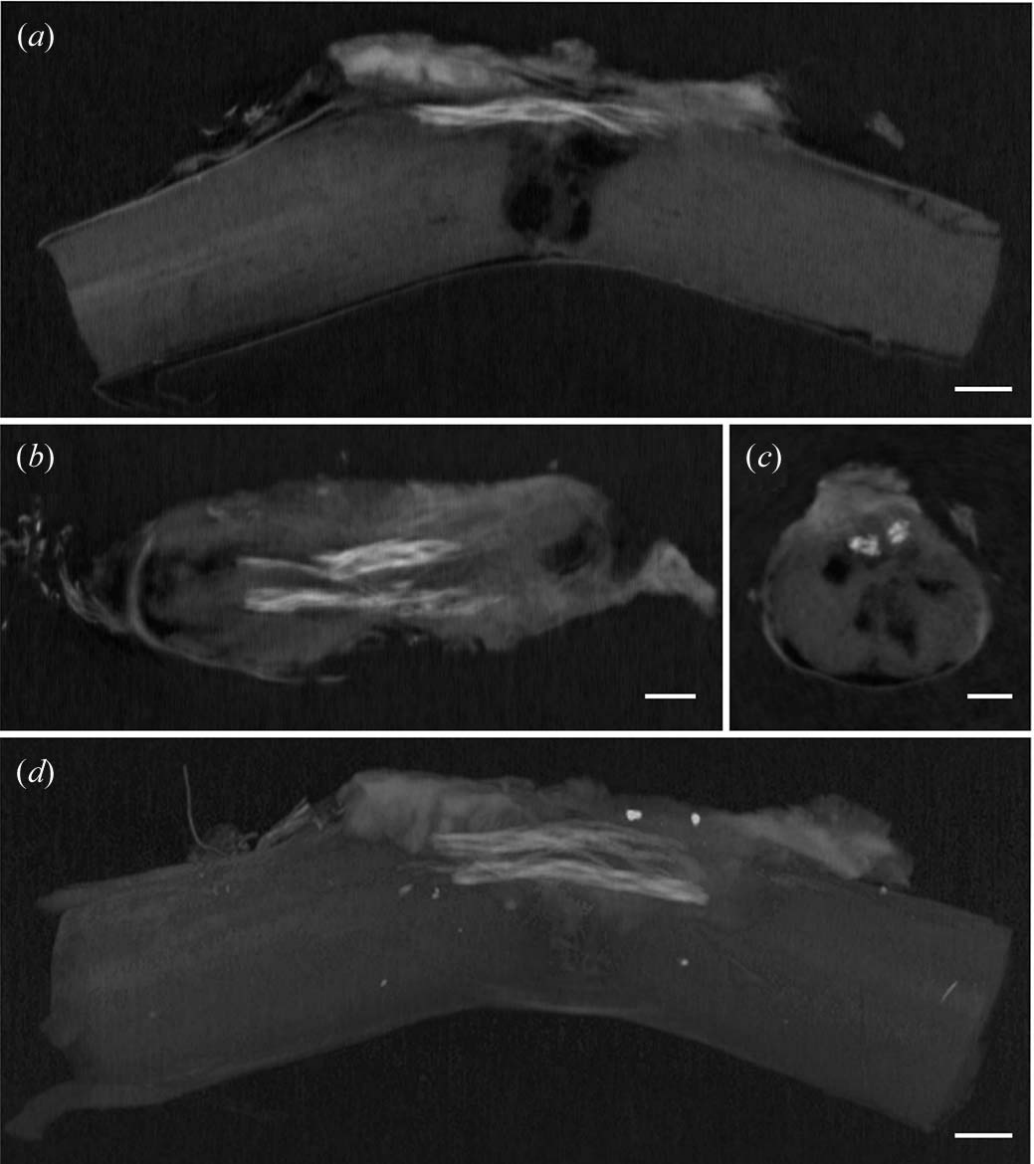
Microstructure of braided scaffolds in the injured spinal cord was visible on X-ray phase-contrast CT with a synchrotron radiation source. (*a*–*c*) X-ray phase-contrast CT images of the injured spinal cord 28 days after scaffold implantation in sagittal (*a*), horizontal (*b*) and coronal slices (*c*). Note the preserved tubular structure of the braided scaffold. (*d*) A three-dimensional image rendering of the injured spinal cord 28 days after scaffold implantation. Scale bar = 500 µm.

**Figure 5:**
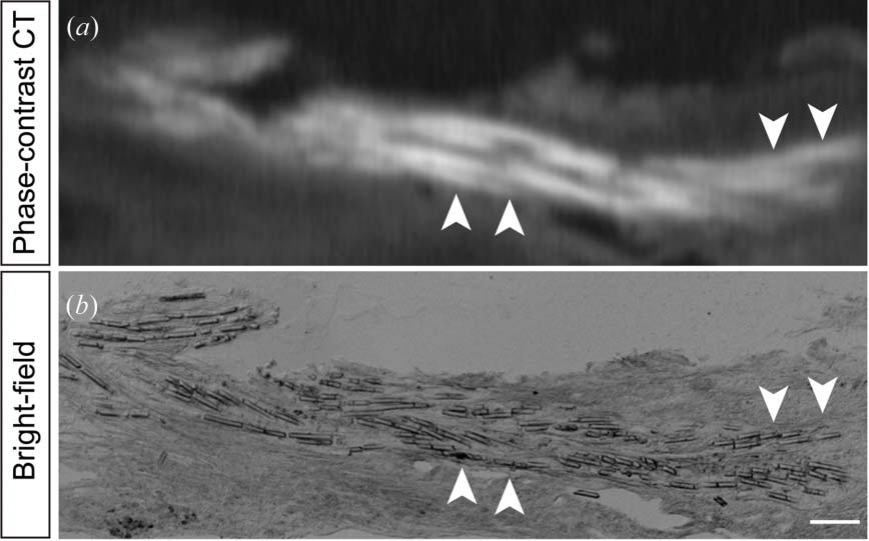
PGA microfibres of braided scaffolds in the injured spinal cord were visible on X-ray phase-contrast CT with a synchrotron radiation source. High-magnification images of the X-ray phase-contrast CT (*a*) and histological section (*b*) in the sagittal plane 28 days after scaffold implantation into injured spinal cord. Arrowheads indicate PGA microfibres that were clearly visible using the X-ray phase-contrast imaging. Scale bar = 50 µm.

We applied X-ray phase-contrast CT imaging based on a Talbot grating interferometer (Fig. 2 ▶). Raw images observed with the X-ray Talbot grating interferometer were five-step fringe-scan images observed without moiré fringes (Fig. 3*a* ▶). A phase-retrieval method and a flat-field correction were used to obtain a differential phase-shift image of the sample (Fig. 3*b* ▶), and one-dimensional integration was performed to obtain the phase-shift image (Fig. 3*c* ▶). In the phase-contrast CT measurement, phase-contrast slice images were reconstructed from 600 projection images. Phase-contrast reconstructed images clearly depicted the two braided scaffolds in sagittal, horizontal and coronal slices 28 days after implantation into the injured spinal cord [Figs. 4(*a*)–4(*c*) ▶]. A three-dimensional rendering of the injured spinal cord after scaffold implantation clearly depicted the continuity of the two braided scaffolds in the injured spinal cord (Fig. 4*d* ▶). In addition, the braided scaffold retained its hollow structure in coronal images (Fig. 4*c* ▶). The X-ray phase-contrast CT image and histological image clearly depicted microfibres of the braided scaffold 28 days after implantation into the spinal cord [Figs. 5(*a*) and 5(*b*) ▶]. These results suggest that X-ray phase-contrast CT imaging can be used to visualize the microstructure of a scaffold, including the hollow tubular structure (about 120 mm in diameter) and PGA microfibres.

### 3.2. Quantitative *in situ* analyses of degradation rate after implantation of the braided scaffold into the injured spinal cord   

**Figure 6:**
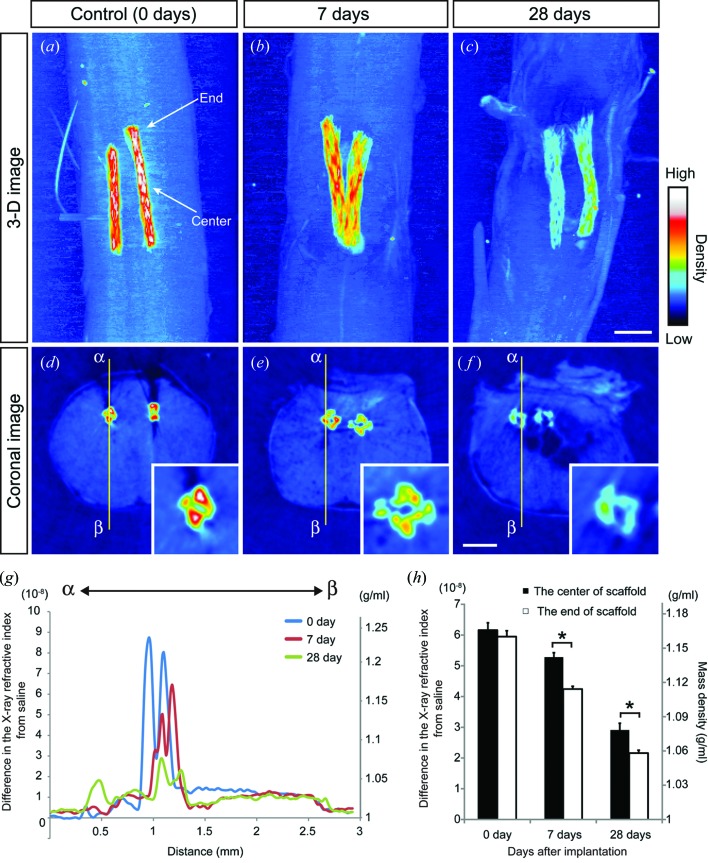
Degradation of braided scaffolds in the injured spinal cord was quantitated by X-ray phase-contrast CT with a synchrotron radiation source. (*a*–*f*) Relative mass density maps acquired with X-ray phase-contrast CT on 0 (*a*, *d*), 7 (*b*, *e*) and 28 (*c*, *f*) days after implantation. Three-dimensional (*a*–*c*) and coronal two-dimensional (*d*–*f*) images acquired with X-ray phase-contrast CT revealed that the density of the scaffold gradually decreased in the spinal cord. At 7 and 28 days after implantation the density in the central portion of the scaffold was higher than that in the end portion, indicating faster degradation in the peripheral portion. Centre and end labels in (*a*) represent the central and end portions of the scaffold, respectively. (*g*) The X-ray refractive index difference from saline (Δδ value) and estimated mass density through the spinal cord from α to β [described in panels (*d*)–(*f*)] at 0 (blue), 7 (red) and 28 (green) days after implantation. Note the densities of the scaffold gradually decreased in the spinal cord. (*h*) Mass density in the central and end portions of the scaffold after implantation into the spinal cord. *p < 0.01. Values are means ± SEM. Scale bar = 500 µm.

The density map obtained by X-ray phase-contrast imaging based on the Talbot grating interferometer provided information on the relative density of the spinal cord tissue 0, 7 and 28 days after scaffold implantation [Figs. 6(*a*)–6(*f*) ▶]. To quantify the degradation of the biodegradable scaffold in the injured spinal cord we calculated the X-ray refractive index difference (Δδ), which is associated with mass density (ρ) (Momose *et al.*, 1996 ▶; Hoshino *et al.*, 2011 ▶). Refractive index profiles were measured in the coronal plane from α to β [Figs. 6(*d*)–6(*g*) ▶]. Δδ (ρ) of the spinal cord ranged from 0.90 × 10^−8^ (1.02 g ml^−1^) to 1.29 × 10^−8^ (1.03 g ml^−1^) at the ventral level [2–2.5 mm from α; Fig. 6(*g*) ▶]. This is consistent with the density of nerve tissue estimated using crystal-interferometer-based imaging, which ranged from 1.020 to 1.035 g ml^−1^ (Beckmann *et al.*, 1999 ▶).

The mean mass density of the implanted scaffolds was measured at 0, 7 and 28 days after implantation. The mass density (ρ) calculated from the Δδ value (6.05 ± 0.22 × 10^−8^) of the scaffold in the spinal cord at 0 days after implantation was 1.16 ± 0.006 g ml^−1^ (*n* = 6). Δδ of the scaffold decreased in the 28 days after implantation into the spinal cord, and was 4.76 ± 0.12 × 10^−8^ (ρ: 1.13 ± 0.003 g ml^−1^) at 7 days after implantation (*p* < 0.01, *n* = 6) and 2.52 ± 0.13 × 10^−8^ (ρ: 1.07 ± 0.004 g ml^−1^) at 28 days after implantation (*p* < 0.01, *n* = 8). In addition, there was a difference in the relative density of the central and end portions of the scaffold at 7 and 28 days after implantation [Figs. 6(*a*)–6(*c*) ▶]. At day 0 there was no difference in the mass density of the central and end portions of the scaffold, but at 7 and 28 days after implantation the mass density at the end portion of the scaffold was significantly lower than at the central portion (Fig. 6*h* ▶), suggesting that the braided scaffolds gradually degraded from the end to centre in the 28 days after implantation. These results indicate that the degradation profile of braided scaffolds in the spinal cord can be visualized with X-ray phase-contrast CT imaging based on the Talbot grating interferometer with a synchrotron radiation source.

## 4. Discussions   

In recent years a variety of materials have been tested in the development of artificial biosynthetic implants for tissue regeneration after spinal cord injury. The environment of the injured spinal cord expresses inhibitory molecules both in the extracellular matrix and in adult myelin that limit axonal plasticity, and fails to express growth factors in appropriate spatial and temporal gradients to stimulate axonal growth (Silver & Miller, 2004 ▶; Yiu & He, 2006 ▶; Rolls *et al.*, 2009 ▶). Axonal growth is supported by inherent properties of the selected polymer, the architecture of the scaffold, permissive microstructures and surface modifications to provide improved adherence and growth directionality. To promote directional axon regrowth after SCI, many scaffolds have microstructure such as pores (Thomas *et al.*, 2013 ▶), grooves (Goldner *et al.*, 2006 ▶), capillary (Prang *et al.*, 2006 ▶) or polymer fibres (Yao *et al.*, 2009 ▶; Hurtado *et al.*, 2011 ▶). Thus, it is important to visualize the microstructures of implanted scaffold using biomedical imaging to precisely evaluate neural tissue repair. Several studies have reported that MRI can be used to visualize implanted scaffolds within the spinal cord in three dimensions (Prang *et al.*, 2006 ▶; Rooney *et al.*, 2008 ▶), but MRI cannot effectively characterize three-dimensional microstructure due to limitations in temporal and spatial resolution. For example, 17.6 T MRI could detect alginate-based highly anisotropic capillary hydrogels (a 0.5 × 0.5 × 1 mm block) in the injured spinal cord, but not the microstructure (capillaries with 27 ± 4 µm diameter) of the scaffold (Prang *et al.*, 2006 ▶). In the present study, X-ray phase-contrast CT images clearly visualized braided scaffold (a 0.15 × 0.15 × 2 mm tube) in the injured spinal cord and, surprisingly, also visualized some bundles of PGA fibres (single-fibre diameter: 10 µm), which is the microstructure of the braided scaffold. X-ray phase-contrast CT imaging has the potential to detect various scaffold microstructures *in situ* after implantation into an injured spinal cord.

Biodegradable polymers are an important material for tissue engineering. In the present study the braided scaffolds were fabricated from PGA polymer. PGA is degraded by random hydrolysis and is also metabolized by enzymes such as esterases *in vivo*. The degradation product, glycolic acid, can enter the tricarboxylic acid cycle and is finally excreted as water and carbon dioxide (Gunatillake & Adhikari, 2003 ▶). Although degradation profiles of PGA threads in phosphate buffer have been reported at 310 K (Chu, 1981*a* ▶,*c* ▶), PGA degradation in the central nervous systems has not been investigated. Three-dimensional X-ray phase-contrast CT images revealed that the braided scaffold gradually degraded from the end to the centre of the scaffold in the 28 days after implantation into the injured spinal cord. The refractive index of the braided scaffold in the injured spinal cord decreased by 58.4% in the 28 days after implantation. The reported mass loss of PGA sutures *in vitro* was around 42% with complete loss of mechanical properties at 49 days after incubation (Chu, 1981*b* ▶; Gunatillake & Adhikari, 2003 ▶). The degradation rate of the scaffold in the injured spinal cord in the present study was faster than that of PGA sutures observed in distilled water at 310 K, and this may be due to hydrolytic enzyme activity of macrophages. Previous studies have indicated that there is synthesis and release of hydrolytic enzymes with a strong degradative potential during macrophage contact with polyurethanes (Labow *et al.*, 2001 ▶). After SCI, macrophages invade the lesion and the areas of greatest glial scarring correlate well with the largest numbers of activated macrophages (David & Kroner, 2011 ▶). These results suggest that, in the present study, activated macrophages gathered around implanted braided scaffold and released hydrolytic enzymes. Thus, the degradation rate was faster *in vivo* than *in vitro*. Furthermore, the degradation rate at the end of the braided scaffold (near the glial scar) was faster than at the centre of the braided scaffold (the core of the lesion) at 7 and 28 days after implantation, which is consistent with the release of hydrolytic enzymes from large numbers of activated macrophages near the glial scar. These results indicate that the advantages of phase-contrast CT over previous methods of degradation evaluation, such as visual observation, weight loss measurement and changes in mechanical properties, are quantitative density localization of scaffolds in the tissue.

Several kinds of X-ray phase-contrast imaging methods have been developed, including crystal interferometer, analyzer-based imaging (Bonse & Hart, 1965 ▶; Momose *et al.*, 1996 ▶), propagation-based imaging (Snigirev *et al.*, 1995 ▶; Wilkins *et al.*, 1996 ▶; Cloetens *et al.*, 1999 ▶) and grating-based imaging (Momose *et al.*, 2003 ▶; Hoshino *et al.*, 2011 ▶, 2012 ▶). Crystal-interferometer-based phase-contrast imaging techniques have ultra-high sensitivity for phase objects including biological samples, and phase-contrast tomography enables observation of slight density differences in biological soft tissues. In fact, crystal-interferometer-based phase-contrast images can successfully visualize the microstructure of various tissues (Momose *et al.*, 1996 ▶; Beckmann *et al.*, 1999 ▶; Shinohara *et al.*, 2008 ▶; Connor *et al.*, 2009 ▶; Wu *et al.*, 2009 ▶). However, this system requires highly brilliant X-rays, such as synchrotron radiation (Shinohara *et al.*, 2008 ▶; Wu *et al.*, 2009 ▶). On the other hand, an approach using the grating interferometer permits the use of conventional X-ray sources in the field of joint disease (Tanaka *et al.*, 2013 ▶). Thus, X-ray phase-contrast imaging based on the Talbot grating interferometer represents a break-through for clinical diagnosis after scaffold implantation in various tissues.

## 5. Conclusions   

In conclusion, X-ray phase-contrast imaging based on the Talbot grating interferometer was able to detect polymer-based scaffolds and their microstructure after implantation into the injured spinal cord and was able to quantitatively determine the biodegradable profile after implantation. This approach will allow the precise monitoring of implanted biodegradable scaffolds that have microstructures to promote new tissue generation and functional recovery after SCI. This method is relevant not only to SCI but to any pathology after implantation of biodegradable polymer scaffolds. X-ray phase-contrast imaging based on the Talbot grating interferometer can be performed on various soft tissues and represents a versatile technique for a broad range of preclinical applications in tissue engineering strategies.
